# Microglial Pro-Inflammatory and Anti-Inflammatory Phenotypes Are Modulated by Translocator Protein Activation

**DOI:** 10.3390/ijms20184467

**Published:** 2019-09-10

**Authors:** Eleonora Da Pozzo, Chiara Tremolanti, Barbara Costa, Chiara Giacomelli, Vladimir M. Milenkovic, Stefanie Bader, Christian H. Wetzel, Rainer Rupprecht, Sabrina Taliani, Federico Da Settimo, Claudia Martini

**Affiliations:** 1 Department of Pharmacy, University of Pisa, 56126 Pisa, Italy; eleonora.dapozzo@unipi.it (E.D.P.); chiara.tremolanti@phd.unipi.it (C.T.); chiara.giacomelli@unipi.it (C.G.); sabrina.taliani@unipi.it (S.T.); federico.dasettimo@unipi.it (F.D.S.); claudia.martini@unipi.it (C.M.); 2 Department of Psychiatry and Psychotherapy, Molecular Neurosciences, University of Regensburg, 93059 Regensburg, Germany; vladimir.milenkovic@ukr.de (V.M.M.); stefanie.bader@stud.uni-regensburg.de (S.B.); christian.wetzel@klinik.uni-regensburg.de (C.H.W.); rainer.rupprecht@medbo.de (R.R.)

**Keywords:** translocator protein 18 KDa, human microglial cells, neuroinflammation, interleukins, neurosteroids

## Abstract

A key role of the mitochondrial Translocator Protein 18 KDa (TSPO) in neuroinflammation has been recently proposed. However, little is known about TSPO-activated pathways underlying the modulation of reactive microglia. In the present work, the TSPO activation was explored in an in vitro human primary microglia model (immortalized C20 cells) under inflammatory stimulus. Two different approaches were used with the aim to (i) pharmacologically amplify or (ii) silence, by the lentiviral short hairpin RNA, the TSPO physiological function. In the TSPO pharmacological stimulation model, the synthetic steroidogenic selective ligand XBD-173 attenuated the activation of microglia. Indeed, it reduces and increases the release of pro-inflammatory and anti-inflammatory cytokines, respectively. Such ligand-induced effects were abolished when C20 cells were treated with the steroidogenesis inhibitor aminoglutethimide. This suggests a role for neurosteroids in modulating the interleukin production. The highly steroidogenic ligand XBD-173 attenuated the neuroinflammatory response more effectively than the poorly steroidogenic ones, which suggests that the observed modulation on the cytokine release may be influenced by the levels of produced neurosteroids. In the TSPO silencing model, the reduction of TSPO caused a more inflamed phenotype with respect to scrambled cells. Similarly, during the inflammatory response, the TSPO silencing increased and reduced the release of pro-inflammatory and anti-inflammatory cytokines, respectively. In conclusion, the obtained results are in favor of a homeostatic role for TSPO in the context of dynamic balance between anti-inflammatory and pro-inflammatory mediators in the human microglia-mediated inflammatory response. Interestingly, our preliminary results propose that the TSPO expression could be stimulated by NF-κB during activation of the inflammatory response.

## 1. Introduction

The translocator protein 18 KDa (TSPO) exhibits ubiquitous localization in multicellular organisms and shows an evolutionary conservation, which are all characteristics that suppose its involvement in fundamental biological processes [1,2,3]. Among these, biological responses to inflammation have been particularly investigated. Following neuronal damage, TSPO expression increases in glial cells, the nervous system cells mainly deputized to immune surveillance, and the system returns to normal levels at the resolution of the phenomenon [4].

Glia is composed of various types of cell populations, including microglia. In the central nervous system, these cells are responsible for the maintenance of a physiological condition and extremely sensitive to small pathological changes [5]. Following brain injury, microglia activate, proliferate, and change their phenotype, which drives the initiation of the inflammation process. The classical activated phenotype, called M1, releases pro-inflammatory molecules such as interleukin (IL) 6, 8, 1β, and Reactive Oxygen Species (ROS) and has been associated with neurotoxicity activity [6]. Conversely, the M2 phenotype is characterized by anti-inflammatory properties, which promotes tissue remodeling and repairing by the release of high levels of IL-4 and IL-10 [6]. Classical M1 microglia activation and proliferation occur in almost all neurodegenerative pathologies of the brain [7,8]. The microglia can shift from M1 to M2 phenotype in particular conditions and contribute to neuroprotection. Therefore, understanding the modulation of the microglia phenotypic shift, could be crucial to develop strategies for treating neurodegenerative disorders.

The evidence of TSPO upregulation in inflamed microglia suggests its promising use as a “molecular sensor” of neuronal damage and repair, as well as an emerging neuroinflammation target for a positron emission tomography scan [9]. However, the functional consequences underlying the variations of TSPO expression levels are not yet known. It was supposed that TSPO induces the production of neurosteroids locally in the damaged area, which modulate the glia or neuron activities, and promote neuronal survival [10]. Indeed, the TSPO is an integral component of the outer mitochondrial membrane that binds the first substrate of neurosteroidogenesis cholesterol with nanomolar affinity in a region containing a cholesterol recognition aminoacidic consensus (CRAC) domain [11]. Moreover, TSPO has been proposed to be part of the steroidogenic transduceosome/metabolome, which is a multiprotein machinery that controls the first steps of neurosteroidogenesis [2,12,13]. TSPO is considered a multifaced protein as it interacts dynamically with other mitochondrial proteins. For example, with the voltage dependent anion channel (VDAC), it modulates a number of additional mitochondrial activities, including respiration, porphyrin binding/heme biosynthesis, permeability transition pore opening, and production of reactive oxygen species [14].

Several studies supporting the involvement of TSPO in neuroinflammation have applied synthetic TSPO ligands as a tool to activate the protein in both in vivo and in vitro models of neuroinflammation/neurodegeneration [15,16,17,18,19,20,21]. Such studies have been mainly performed in rodents and have accumulated heterogeneous data, which suggests that this pharmacological approach requires further information before proposing a mechanistic model. Since TSPO discovery, several selective and high-affinity ligands have been developed. Among the first selective TSPO ligands, the 5-chlorine derivative of diazepam Ro5-4864 and the isoquinoline PK11195 are considered classical TSPO ligands. All the newly introduced synthetic ligands have shown high affinity at Ro5-4864 or PK1195 binding sites. We have recently found that the TSPO ligand ability to stimulate neurosteroidogenesis is linked to the time in which a ligand remains bound to the PK11195 or Ro5-4864 site (a parameter called “Residence Time”—RT), irrespective of ligand binding affinity. In particular, a ligand with a RT value of at least 100 min to PK11195 site or at least 50 min to Ro5-4864 site stimulates efficaciously the neurosteroidogenic function of TSPO [22,23,24,25,26]. Noteworthy, in rat astrocytes, a long interaction of ligands at the PK11195 site stimulates effectively TSPO neurosteroidogenic function promoting cellular well-being and counteracting an excessive neuroinflammatory response [26,27]. Further investigations are certainly needed in human experimental models due to the differences between human and rodent glia, which are emerging both in biochemistry and in response to pharmacological substances [28].

Herein, in order to deepen the knowledge about the role of TSPO in the neuroinflammatory response, the effects exerted by amplification or attenuation of the physiological function of TSPO on the release of pro-inflammatory and anti-inflammatory interleukins were investigated in an in vitro model of inflamed human primary microglia. In the case of the amplification experimental approach, the effects induced by TSPO stimulation with ligands, characterized by low and high neurosteroidogenic capacity, were compared.

## 2. Results

### 2.1. Human Microglial Inflammation: In Vitro Model Setting

The immortalized human microglia C20 cells, which respond to immunogenic stimulation similarly to the primary human microglia [29], were employed as an in vitro model. It has been reported that human microglia respond to different stimuli with respect to the rodent microglia. For example, bacterial lipopolysaccharide (LPS) and interferon-γ (INF-γ) are able to induce inflammatory signaling in both human and rodent microglia [30,31]. Conversely, only the human microglia are susceptible to the treatment with Interleukin-1 beta (IL-1β) [31,32,33]. The use of different pro-inflammatory stimuli could activate specific intracellular pathways highlighting diverse responses of microglia. Herein, two in vitro settings of inflammatory activation by immunogenic stimuli were chosen. The first was obtained following exposure of C20 cells to IL-1β (20 ng/mL), which is considered the master regulator of neuroinflammation. It is released by microglial cells and astrocytes (as the main source) and regulates its further release in an autocrine/paracrine manner [33]. The second was obtained through the simultaneous exposure of C20 cells to IL-1β (100 ng/mL) and another soluble cytokine exerting an important role in the human microgliosis, the INF-γ (50 ng/mL) [34]. The use of a combined stimulus at high concentrations could mimic an exaggerated inflammatory response. The choice of these immunogenic stimuli was also supported by previous data suggesting that the activated C20 cells upregulate the gene transcription of the INF-γ response, and the nuclear factor kappa-light-chain-enhancer of activated B cells (NF-κB) signaling. The latter is considered the main pathway activated by IL-1β [29].

**Figure 1:**
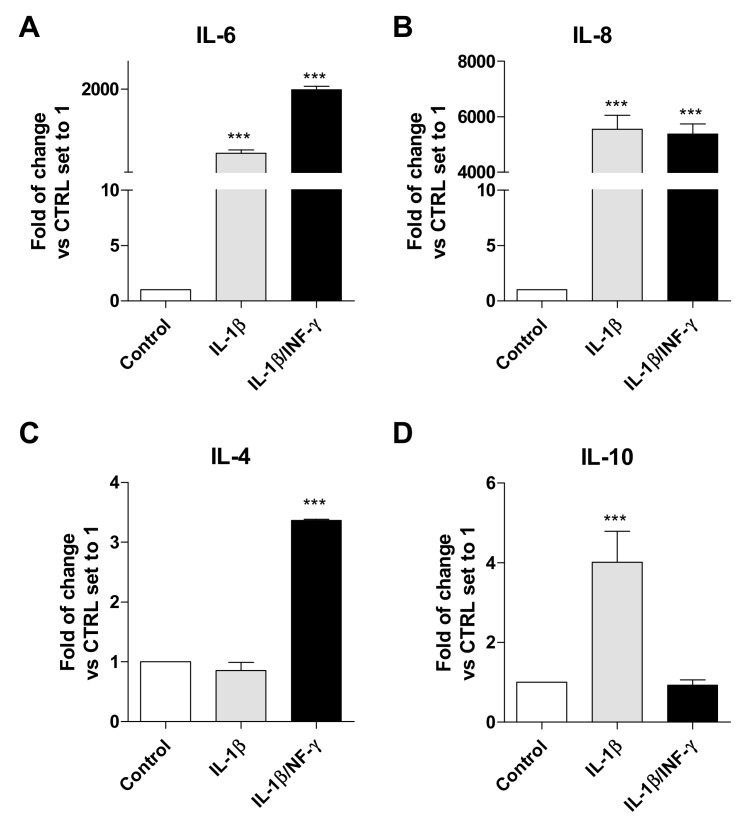
Interleukin production by C20 cells as an inflamed model of human microglia. The mRNA levels of the pro-inflammatory interleukins IL-6 and IL-8 (**A**,**B**) and the anti-inflammatory interleukins IL-4 and IL-10 (**C**,**D**) were monitored in C20 cells following IL-1β (20 ng/mL, grey bars) and IL1-β/INF-γ (100 ng/mL/50 ng/mL black bars) treatment for 24 h. The data are expressed as the fold of change *versus* the control, which was set to 1, and are presented as the mean values ± SEM of two independent experiments performed in duplicate. The significance of the differences was determined by one-way ANOVA, which was followed by Bonferroni’s post-test: *** *p* ≤ 0.001, vs. control.

**Figure 2:**
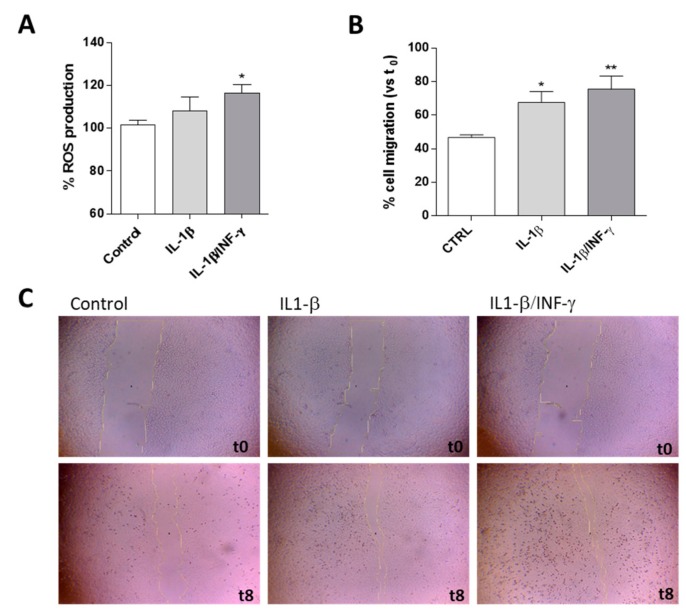
Effects of IL-1β or IL1-β/INF-γ on C20 cell ROS production and migration ability. C20 cells were treated with IL-1β (20 ng/mL) or IL1-β/INF-γ (100 ng/mL/50 ng/mL) for 24 h. (**A**) ROS generation was quantified and reported as a percentage with respect to untreated cells (Control). Each bar represents the mean ± SEM of three replicates from two independent experiments. (**B**) C20 cells were treated as reported and the percentage of gap closure with respect to the evaluated t_0_. The data are presented as the mean values ± SEM of at least two independent experiments performed in duplicate (4·×·magnification). (**C**) Representative images of the scratch wounds at t = 0 h and t = 8 h are shown. The significance of the differences was determined by one-way ANOVA, followed by Bonferroni’s post-test: * *p* ≤ 0.05, ** *p* ≤ 0.01 vs. control.

The cytokine transcription levels, migration ability, and ROS production were assessed following 24 h of IL-1β or IL-1β/INF-γ exposure (Figure 1 and Figure 2).

For both immunogenic stimuli, the results showed a statistically highly significant increase in the transcripts of the pro-inflammatory IL-6 (Figure 1A, *p* < 0.001) and IL-8 (Figure 1B, *p* < 0.001). Both stimuli promoted a higher transcription of IL-8 with respect to IL-6. Among the several inflammatory molecules analyzed in a very recent work, IL-8 resulted in a highly-induced cytokine in C20 cells when subjected to another immunogenic stimulus (Tumor necrosis factor-α) [29]. Together, these results suggest that IL-8 is one of the main pro-inflammatory mediators activated by reactive C20 cells, which is in agreement with the typical characteristics observed for human primary microglia [35].

Challenging C20 cells with the single or combined inflammatory stimulus produced different responses for the transcription of the anti-inflammatory interleukin IL-4. A significant increase in IL-4 (Figure 1C, *p* < 0.001) was shown following exposure to IL-1β/INF-γ. Conversely, an increase in IL-10 (Figure 1D, *p* < 0.001) was observed following exposure to IL-1β. Actually, the cytokine secretion profile is regulated by a complex control machinery to maintain a proper balance among pro-inflammatory and anti-inflammatory mediators [36]. The observed increases of anti-inflammatory molecules were much lower than those observed for the pro-inflammatory interleukins.

The activation of microglia is dependent upon a number of features, not the least of which is the production of reactive oxygen species (ROS) [37]. Challenging C20 cells with IL-1β or IL-1β/INF-γ caused an increase of ROS levels (Figure 2A). However, only the combined stimulus produced a statistically significant increase in ROS production (116.5·± 3.9 % vs. CTRL *p* < 0.05) compared to the control cells. This result was in agreement with literature data suggesting that the human inflamed microglia, unlike those of the rodent, do not secrete large quantities of ROS [28].

To assess the effect of IL-1β or IL-1β/INF-γ on microglial motility, normally increased in activated microglia, a scratch wound assay was performed (Figure 2B,C). As shown in Figure 2C, representative microscopic images clearly illustrate that the inflamed-C20 cells exhibited an enhanced migratory potential when compared with the control. In particular, both the single and the combined stimuli were able to significantly enhance gap closure with respect to the control (*p* < 0.001). These data are in accordance with the ability of activated microglia to increase cell motility [38,39].

**Figure 3:**
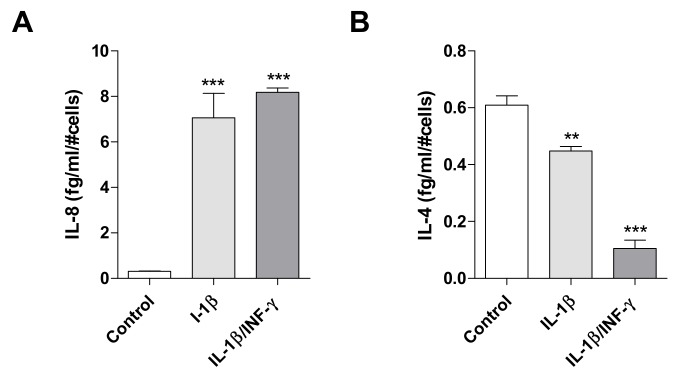
Effects of inflammatory stimuli on release of IL-8 and IL-4 from C20 cells. The pro-inflammatory IL-8 (**A**) and anti-inflammatory IL-4 levels (**B**) were evaluated in conditioned medium derived by C20 cells treated with IL-1β (20 ng/mL) or IL1-β/INF-γ (100 ng/mL/ 50 ng/mL) for 24 h. The amount of interleukins measured was normalized to the number of cells and expressed as fg/mL/#cells. The bars represent the mean ± SEM of two different experiments performed in triplicate. The significance of the differences was determined by one-way ANOVA, followed by Bonferroni’s post-test: ** *p* ≤ 0.01, *** *p* ≤ 0.001, vs. control.

Based on previous data suggesting an important role of IL-8 in the human reactive microglia, this molecule was chosen as an example of pro-inflammatory interleukin and monitored in the following experiments (Figure 3A). In parallel, IL-4 was chosen as an example of anti-inflammatory interleukin, as it is a key pleiotropic cytokine regulating the brain homeostasis [40]. Importantly, an interplay between the release of IL-4 and IL-8 has been previously observed in human microglia [41].

Following exposure with IL-1β or IL-1β/INF-γ, C20 cells significantly increased the release of IL-8 (*p* < 0.001, Figure 3A), in accordance with the observed increase of its mRNA levels (Figure 1B). Moreover, a significant decrease of IL-4 release was shown (*p* < 0.001, Figure 3B), even if the above results (Figure 1C) showed a simultaneous increase of IL-4 mRNA for the IL-1β/INF-γ sample. The discrepancy between protein levels and their coding transcripts is a frequently observed phenomenon and is often ascribed to the different mechanisms that regulate transcription, translation, and release of the protein from the intracellular pools [42].

### 2.2. The Pharmacological Stimulation of TSPO Attenuates the Inflamed Phenotype of C20 Cells

**Figure 4:**
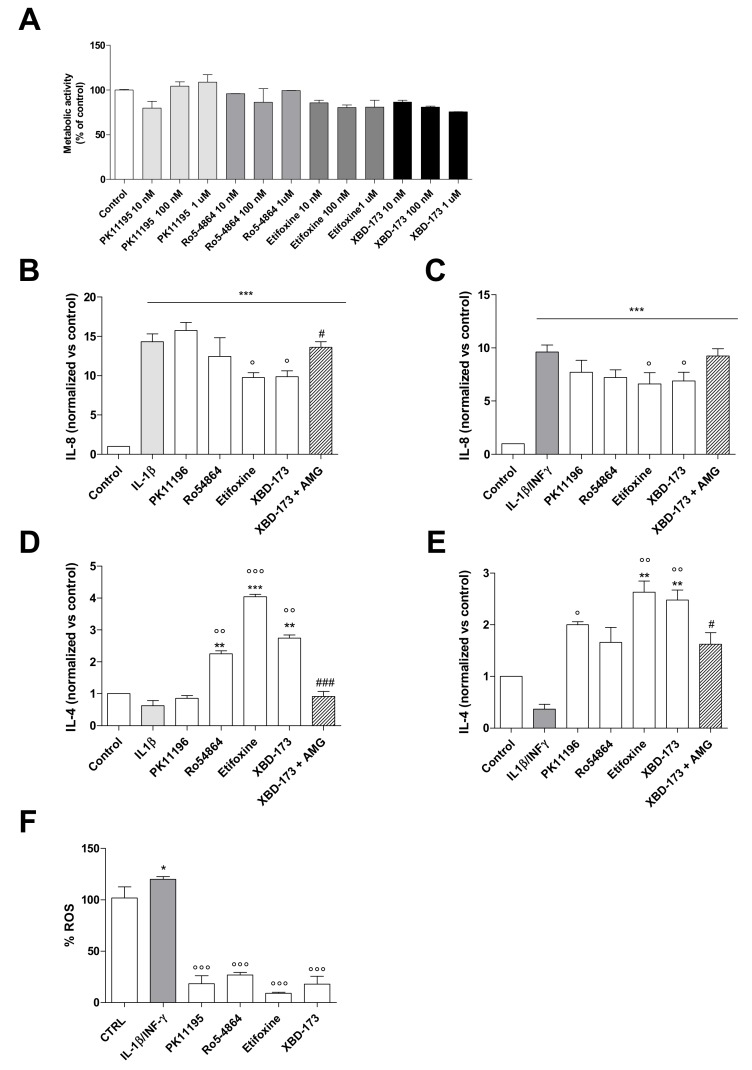
Effects of TSPO pharmacological stimulation on cell viability of C20 cells and interleukin release from inflamed C20 cells. (**A**) TSPO ligands’ effects on C20 cell viability. Cells were treated with three different concentrations of PK11195, Ro5-4864, XBD-173, or Etifoxine (10 nM, 100 nM, and 1 μM) in serum-free medium. The MTS assay was performed after 24 h of treatment. Data are expressed as percentages of cell viability compared to the control, which was set to 100%. No significant differences were observed. (**B**–**D**) C20 cells were pre-treated with TSPO ligands (100 nM) for 2 h in serum-free medium. Then, IL-1β (20 ng/mL) or IL1-β/INF-γ (100 ng/mL/ 50 ng/mL) was added. In the case of treatment with XBD-173, samples of the C20 cells were in parallel pre-treated with AMG (50 µM) 1 h before the addition of the TSPO ligand. After 24 h, the IL-8 and IL-4 release was evaluated. In particular, the figure shows the release of IL-8 in the IL-1β-inflamed sample (**B**), IL-8 in the IL-1β/INF-γ-inflamed sample (**C**), IL-4 in the IL-1β-inflamed sample (**D**), and IL-4 in the IL-1β/INF-γ-inflamed sample (**E)**. The concentration of interleukins was obtained by the enzyme-linked immunosorbent assay (ELISA) and normalized to the amount of cytokine in untreated cells set to 1. The bars represent the mean ± SEM of at least three duplicates from three different experiments. (**F**) C20 cells were treated as above and ROS generation was evaluated. Data are expressed as percentages with respect to control, which was set to 100%. Each bar represents the mean ± SEM of three replicates from two independent experiments. The significance of the differences was determined by one-way ANOVA, which was followed by Bonferroni’s post-test or student *t*-test: * *p* ≤ 0.05, ** *p* ≤ 0.01, *** *p* ≤ 0.001, vs. control, ° *p* ≤ 0.05, °° *p* ≤ 0.01, °°° *p* ≤ 0.001, vs. IL-1β or IL1-β/INF-γ, ^#^ *p* ≤ 0.05, and ^###^ *p* ≤ 0.001 vs. XBD-173.

The effect of TSPO ligands on C20 cell viability was investigated (Figure 4A) for the first time. Ligand increasing concentrations (10 nM, 100 nM and 1 μM) did not cause significant differences between TSPO ligand-treated and control C20 cells. Herein, the 100 nM concentration was chosen as explorative treatment in IL-8 and IL-4 release experiments, because it has been commonly employed in previous studies investigating TSPO effects [43,44,45]. To this aim, C20 cells were treated with TSPO ligands (100 nM) 2 h before the administration of the inflammatory stimulus (IL-1β or IL-1β/INF-γ) for 24 h.

Concerning IL-8, the classical ligands PK11195 and Ro5-4864 did not give statistically significant results. However, a trend toward a decrease in the IL-8 release was observed, above all for Ro5-4864 (Figure 4B,C). Conversely, the significant increase of its release induced by IL-1β and IL-1β/INF-γ was significantly counteracted by the pre-treatment with the highly steroidogenic compound Etifoxine and XBD-173 (*p* < 0.05, Figure 4B,C).

The classical ligands were able to differently increase IL-4 release in the two inflammatory models (Figure 4D,E). In particular, Ro5-4864 was able to increase the IL-4 levels under both the inflammatory stimuli (in a statistically significant manner following IL-1β treatment and near significance under IL-1β/INF-γ inflammation). Conversely, PK11195 increased IL-4 release only after IL-1β/INF-γ stimulus. Differences for the effects induced by PK11195 and Ro5-4864 have been frequently observed in various experimental models, including neuroinflammation [19,21]. A significant increase of IL-4 levels was found following the pre-treatment with Etifoxine and XBD-173 (*p* < 0.001, Etifoxine, *p* < 0.01, XBD-173, Figure 4D,E).

The used TSPO ligands could be divided into two groups: poorly neurosteroidogenic ligands (PK11195 and Ro5-4864) and highly neurosteroidogenic ones (XBD-173 and Etifoxine) [22,23,25]. Moreover, they all are selective for TSPO, with the exception of Etifoxine, which binds with low affinity to the GABA_A_ receptor too [46].

To assess whether the observed effects on interleukin release were mediated by neurosteroids, the cell pretreatment with aminoglutethimide (AMG), which is a synthetic molecule that inhibits the first enzyme of the steroidogenic cascade cytochrome P450 side-chain cleavage (P450scc), was conducted (Figure 4B,E). Despite the similar effects obtained by XBD-173 and Etifoxine in the above experiments, such an evaluation was performed for XBD-173 because of its TSPO selectivity. The steroidogenesis inhibitor AMG was able to counteract the effects of restoring the IL-8 production (Figure 4B) and reducing the secretion of IL-4 (Figure 4D,E). These data indicate that XBD-173 effects are mainly mediated by neurosteroids production. However, AMG did not completely abolish the effect induced by XBD-173 in the samples inflamed with the combined stimulus (Figure 4C,E). Such a result suggests that the release of IL-8 and IL-4 may be influenced in part by additional TSPO activities. In agreement with this, it has been recently shown that XBD-173, in addition to effectively stimulate steroidogenesis, exerts effects on other basic mitochondrial functions, such as oxidative phosphorylation, mitochondrial membrane potential, and calcium homeostasis in murine inflamed microglia [47].

Another effect observed when TSPO was pharmacologically stimulated in inflamed C20 cells concerns the reduction of intracellular ROS levels. All the TSPO ligands were able to significantly counteract the production of ROS mediated by the combined stimulus (*p* < 0.001, Figure 4F), which agrees with the data documented in rodent microglia [48]. TSPO ligands were able to decrease the ROS production under the control levels confirming the role of TSPO in the control of microglia activation (Figure 4F). The same reduction in ROS levels induced by poorly and highly steroidogenic TSPO ligands suggests that this effect cannot be attributed to eventual neurosteroid-dependent pathways. The observed effect is likely due to modulation phenomena triggered by other mitochondrial activities in which TSPO is involved. In general, the documented TSPO activities influencing the ROS production include the modulation of the mitochondrial calcium signaling [49,50], and the transport of the precursor of heme (protoporphyrogen IX), with this latter being an important cofactor for the molecules mainly involved in the ROS production (the cytochromes of the electron transport chain) and for antioxidant enzymes [51]. To the best of our knowledge, at the moment, little is known about the mechanism by which TSPO could exert this modulatory activity on ROS production in microglia. Suggestive hypotheses have been addressed to report a linkage between TSPO and both the entry of calcium into the mitochondria and the production of cyclooxygenase-2 (COX2) [52,53]. In literature, a protective role of TSPO against ROS-induced damage has been demonstrated in various experimental models [50,54,55].

The TSPO ligand nanomolar concentration used in the present experiments corresponds to that employed in previous works investigating the TSPO-mediated pro-life effects [43,44,45]. However, it is important to note that TSPO ligands have contrasting effects based on the used concentration. In fact, micromolar concentrations of high-affinity TSPO ligands have often induced opposite effects with respect to those documented with nanomolar concentrations [56,57,58,59]. However, the mechanisms at the basis of these observed effects is not yet clarified. Interestingly, it could be supposed that the use of the low affinity TSPO ligand, Etifoxine, may avoid the commonly observed negative effects documented with high doses of high-affinity TSPO ligands. However, further studies are needed to provide insights on this issue.

### 2.3. TSPO Knockdown Amplifies C20 Cell Responsiveness to the Inflammatory Stimulus 

**Figure 5:**
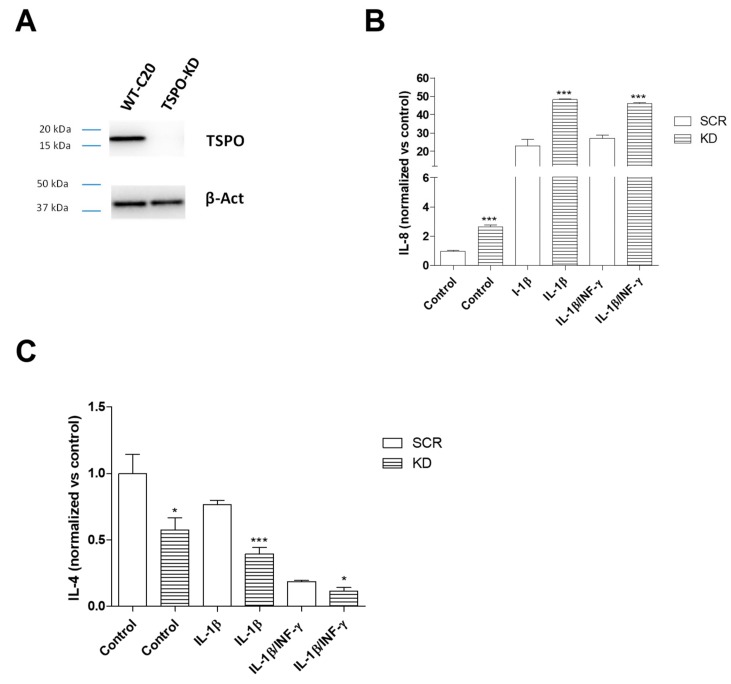
Effects of TSPO knockdown (KD) on interleukin release. (**A**) Representative images of TSPO and β-actin expression on SCR and TSPO KD C20 cells by Western blot analysis. SCR and TSPO KD C20 cells were treated with IL-1β (20 ng/mL) or IL1-β/INF-γ (100 ng/mL/50 ng/mL) for 24 h in serum-free medium and pro-inflammatory IL-8 (**B**) and anti-inflammatory IL-4 level (**C**) were evaluated. The concentration of IL-8 and IL-4 released in the medium was normalized to the number of cells in each well and expressed as fg/mL/#cells. The bars represent the mean ± SEM of two different experiments performed in triplicate. The significance of the differences was determined by one-way ANOVA, which was followed by Bonferroni’s post-test: * *p* ≤ 0.05, ** *p* ≤ 0.01, *** *p* ≤ 0.001, respective WT C20 cell treatment. The comparison between the WT C20 cells and SRC C20 cells samples did not show any significant difference, both in the presence and in the absence of inflammatory stimulus.

In the TSPO knockdown (KD) C20 cells, the reduction of TSPO expression was confirmed by Western blot analysis (Figure 5A).

In inflammatory conditions, TSPO KD cells increased the release of IL-8 vs. Scramble (SCR) C20 cells (for IL-1β: *p* < 0.01, for IL-1β/INF-γ: *p* < 0.001) (Figure 5B). Conversely, TSPO KD cells decreased the release of IL-4 (for IL-1β: *p* < 0.001; for IL-1β/INF-γ: *p* < 0.05) (Figure 5C). The observed increase of the pro-inflammatory cytokine and decrease of the anti-inflammatory cytokine suggests that TSPO plays a negative modulatory role on pro-inflammatory signaling during the activation of the process.

In the absence of inflammatory stimulus, the TSPO KD control C20 cells showed higher IL-8 and lower IL-4 release than SCR control C20 cells (Figure 5B,C), which suggests that TSPO is required to maintain the basal controlled release of the pro-inflammatory and anti-inflammatory cytokines.

### 2.4. Regulation of TSPO Transcription in Microglia Following Inflammatory Stimuli

**Figure 6:**
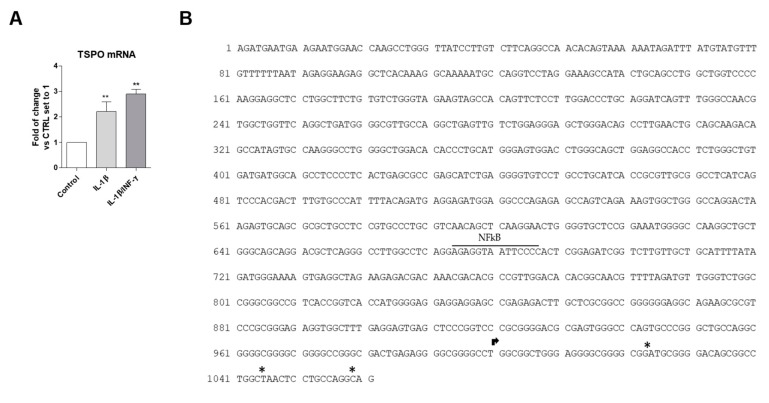
(**A**) TSPO gene expression in WT C20 cells. Cells were treated with IL-1β (20 ng/mL) or IL1-β/INF-γ (100 ng/mL/ 50 ng/mL) for 24 h in serum-free medium and TSPO mRNA transcript levels were assessed by qPCR, and expressed as fold of change versus the control, which was set to 1. Data are presented as the mean values ± SEM of two independent experiments. The significance of the differences was determined by one-way ANOVA, which was followed by Bonferroni’s post-test: ** *p* ≤ 0.01 vs. control. (**B**) The figure shows the proximal promoter of the human *TSPO*, in which the nucleotide sequence for NF-κB is labeled. The arrow indicates the TSS reported on the NCBI site. The asterisks indicate the TSS reported by Batarseh [60].

The levels of TSPO mRNA was evaluated after treatment with both the pro-inflammatory stimuli (Figure 6A). The results showed that the treatment with IL-1β or IL-1β/INF-γ significantly increased the TSPO transcript levels (2.2 ± 0.4-fold of change, IL-1β *p* < 0.01, 2.9 ± 0.2-fold of change IL-1β/INF-γ, *p* < 0.01). 

On the basis of previous data demonstrating that the IL-1β-induced inflammatory signaling leads to the activation of NF-κB in C20 cells [31], the potential presence of the nucleotide sequence for such a transcription factor (TF) was explored in the TSPO proximal promoter. The human TSPO (NCBI ID706) is located from the nucleotide position 43151514 to 43163242 on chromosome 22. Here, the analyzed sequence (1061 nucleotides) includes both the transcription start site (TSS) reported in the NCBI site and the additional TSS recently suggested in various cell lines (located in a window of 40 to 50 bases downstream from the NCBI TSS) [60] (Figure 6B). 

By Transfac analysis, performed by selecting “vertebrate matrices” and the “human promoter” as a background model, the affinity-based ranking of transcription factors (TFs) showed that 30 sequences (Table S1) have significantly more TF binding affinity than we could expect from random sequences in the chosen background set. In particular, the sequence for NF-κB (position 1 of Table S1) was located in the position −312/−325 of the TSPO promoter (numbering with respect to the TSS of the NCBI site) and showed the highest binding affinity (*p* = 0.0068). The PROMO analysis showed that 12 of the 14 nucleotides in the NF-κB sequence correspond to the most probable consensus sequence generated for this TF (dissimilarity 8.39%). The expected random dissimilarity index (RE), which was obtained by performing the match with a random sequence, showed the equal probability value for the four nucleotides (RE equally) of 0.00809 and nucleotide frequency probability in the study sequence (RE query) of 0.01270.

**Figure 7:**
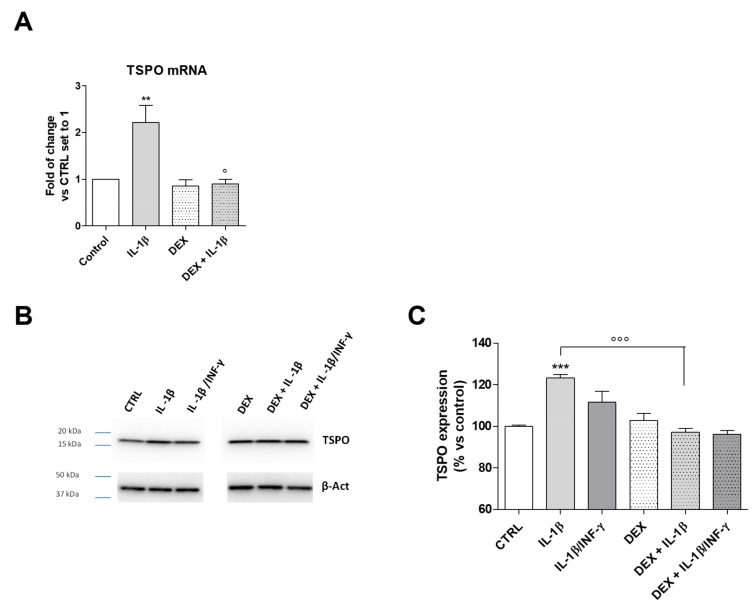
TSPO gene and protein expression in inflamed C20 cells. (**A**) Cells were treated with IL-1β (20 ng/mL) or IL1-β/INF-γ (100 ng/mL/50 ng/mL) for 22 h in serum-free medium and TSPO mRNA transcript levels were quantified and expressed as fold of change versus the control (CTRL), which was set to 1. Data are presented as the mean values ± SEM of two independent experiments performed in duplicate. The C20 cells were treated with IL-1β (20 ng/mL) or IL1-β/INF-γ (100 ng/mL/ 50 ng/mL) for 22 h and TSPO protein expression was determined by Western Blot analysis. Representative images (**B**) and densitometric analysis (**C**) are shown. The data were expressed as the percentage of expression versus control set to 100% and represent the mean values ± SEM of three different experiments. The significance of the differences was determined by one-way ANOVA, followed by Bonferroni’s post-test: ** *p* ≤ 0.01, *** *p* < 0.001 vs. control. ° *p* < 0.05, °°° *p* < 0.001 vs. IL-1β.

It is well known that glucocorticoids exert anti-inflammatory and immunosuppressive activities. It has been demonstrated that the synthetic glucocorticoid dexamethasone (DEX) inhibits the NF-κB activation in different cellular models [61]. The treatment of C20 cells with DEX significantly counteracted the IL-1β-induced TSPO transcription (Figure 7A, *p* < 0.05). This result is in accordance with the presence of the NF-κB nucleotide sequence in the TSPO promoter. The same effects were observed for the protein expression (Figure 7B,C, *p* < 0.001). The treatment with IL-1β/INF-γ increased TSPO mRNA (Figure 6A) and produced only a slight increase in the protein level. INF-γ has been related to proteasome activation and mitophagy induction in diverse immune cells [62]. Thus, the lack of a significant increase of TSPO protein expression could be likely related to the activation of degradative mechanisms.

## 3. Discussion

In response to neuronal injury or infection, the acquisition of complex functional phenotypes allows microglia to participate in the activation and regulation of neuroinflammation. This process is now well recognized as “a double-edged sword” because it can determine beneficial effects as well as detrimental consequences on neurons [63]. The concept and nomenclature of microglial functional phenotypes have been extensively treated by scientific literature [64,65]. However, the physiological fine-tuning of the dynamic changes that bring to their acquisition remains poorly understood, especially in the framework of mechanisms controlling the prevalence of secretion of pro-inflammatory cytokines on anti-inflammatory ones and vice versa. In this context, most information comes from studies conducted on rodent microglia. To the best of our knowledge, little is known about the activity of human microglia, as, increasingly differences are emerging with respect to that of rodents [28].

In the present manuscript, the obtained results are in favor of a homeostatic role for TSPO in the context of dynamic balance between anti-inflammatory and pro-inflammatory mediators in the human microglia-mediated inflammatory response. In our hands, IL-8 and IL-4 (as examples of pro- and anti-inflammatory cytokines, respectively) were studied and their levels were monitored under amplification or reduction of TSPO activity.

The treatment of inflamed C20 cells with TSPO ligands inhibited the secretion of IL-8 and stimulated the release of the IL-4, shifting the dynamic equilibrium of secretion toward an anti-inflammatory profile. The TSPO ligand-induced effects appeared to be mediated by stimulation of the steroidogenesis, as they were abolished when the steroidogenic pathway was enzymatically inhibited. In support of this, neurosteroids have been proven as the most potent endogenous molecules capable of modulating the inflammatory process exerting effects on different cell populations, and, above all, microglia [66]. Notably, it has been reported that some steroids are pro-inflammatory molecules, but the majority of them exert anti-inflammatory effects [67]. In this complex mechanism, the TSPO stimulation triggers and upturns the whole biosynthetic pathway, which causes an overall anti-inflammatory action. Peculiar effects were observed following the treatments with highly and poorly steroidogenic TSPO ligands. Highly steroidogenic ligands (XBD-173 and Etifoxine) attenuated the neuroinflammatory response more effectively than the poorly steroidogenic ones (PK11195 and Ro5-4864), which suggests that the observed modulation on the cytokine release may be influenced by the levels of produced neurosteroids. Probably, an efficient stimulation of steroidogenesis might imply the achievement of neurosteroid levels required to exercise the modulation of microglia activities. In general, our results highlight that the pro-inflammatory cytokine IL-8 secretion was reduced by the highly steroidogenic ligands. Differently, the stimulation of the anti-inflammatory cytokine IL-4 release was induced by both highly and poorly steroidogenic ligands, even though the latter were less effective than the formers. Similar data have been previously obtained in in vitro murine microglia models, in which XBD-173 dampens the microglia reactivity, which reduces the transcript of the pro-inflammatory interleukin IL-6, whereas the poorly steroidogenic ligands PK11195 and Ro5-4864 did not affect the transcription of proinflammatory cytokines, such as TNFα and IL-1β [21,38,48,68]. In addition to the classically known ability of steroids to suppress the multiple inflammatory genes activated in inflammatory diseases [69], some effects induced by steroidogenic TSPO ligands could be explained by the neurosteroid-mediated potentiation of the GABA_A_ receptor function. Indeed, recent literature has reported that the activation of GABA_A_ receptor attenuates the release of proinflammatory cytokines from inflamed microglia [70,71]. In light of this, it should be noted that Etifoxine, beyond its binding to TSPO, can directly interact with the GABA_A_ receptor [43]. Thus, it cannot be excluded that the observed effects can be also triggered by direct interaction with this receptor. However, the concentration of Etifoxine required to functionally activate the GABA_A_ receptor of microglia is much higher (100 μM) [71] than that used in our experimental setting (100 nM).

Opposite results were obtained by the experimental approach in which the function of TSPO was attenuated by silencing of gene expression. When the TSPO KD C20 cells were subjected to exogenous immunogenic stimulation, they exhibited marked responsiveness to the inflammatory stimulus, which shows higher IL-8 and lower IL-4 secretion than wild-type C20 cells. This is in agreement with previous data documented in murine microglia [47,72]. Such a phenomenon was observed for both the in vitro inflammation models investigated. The concomitant increased secretion of the pro-inflammatory cytokine and reduced secretion of the anti-inflammatory cytokine by TSPO KD cells suggests that TSPO is physiologically required for the negative modulation of neuroimmune pro-inflammatory signaling. Interestingly, in the absence of insult, TSPO KD C20 cells appeared unable to maintain the basal controlled release of pro-inflammatory and anti-inflammatory cytokines. A concomitant increase of IL-8 and decrease of IL-4 release was shown in TSPO KD C20 cells, supporting a homeostatic role of TSPO in maintaining their physiological balance.

Herein, another pro-inflammatory signaling parameter explored was the level of the reactive oxygen species. The treatment with PK-11195 has been reported to decrease ROS production in human macrophages [73] and in murine microglia [48]; herein, TSPO ligands were able to modulate the production of ROS in human microglia too. Although the mechanism of TSPO regulation of ROS production is still under debate, the obtained results supports that this ability is not related to steroidogenesis stimulation. Indeed, all tested TSPO ligands were able to reduce effectively and in similar extent the production of ROS in activated microglia, without any relation with their steroidogenic capacity.

The role for TSPO in modulating the neuroinflammatory response is corroborated by the increase in TSPO levels observed in C20 cells inflamed with IL-1β, according to previous data in rodent microglia models [15,38,47,48,72]. It can be hypothesized that the increase in TSPO expression is a physiological tool used by the microglial cell to ensure a controlled release of the pro-inflammatory and anti-inflammatory cytokines. Stimulation of the observed TSPO activity could be mediated by the interaction of the protein with its endogenous ligands, which are known to stimulate steroidogenesis [74], and are produced by steroidogenic cells, including microglia [48].

Based on previous data demonstrating that the IL-1β-induced inflammatory pathway leads to the activation of the transcription factor NF-κB pathway in C20 cells. The potential presence of the NF-κB binding sequence was investigated in the proximal promoter of the human TSPO gene. The NF-κB transcription factor gave the most significant result in terms of predicted binding affinity and binding sequence homology. Interestingly, the sequence for NF-κB was localized in the promoter region that has been recently involved in epigenetic regulation of TSPO [59,75].

Although the in silico result offers a theoretical indication, it allows us to focus the attention on the promoter region with this transcription factor, which could interact and encourages us to undertake further experiments to ascertain the role of NF-κB in the TSPO transcription regulation. Here, the reduction of TSPO levels in inflamed C20 cells were observed following the pharmacological inhibition of the NF-κB pathway with DEX, which provides important evidence to support this hypothesis.

**Figure 8:**
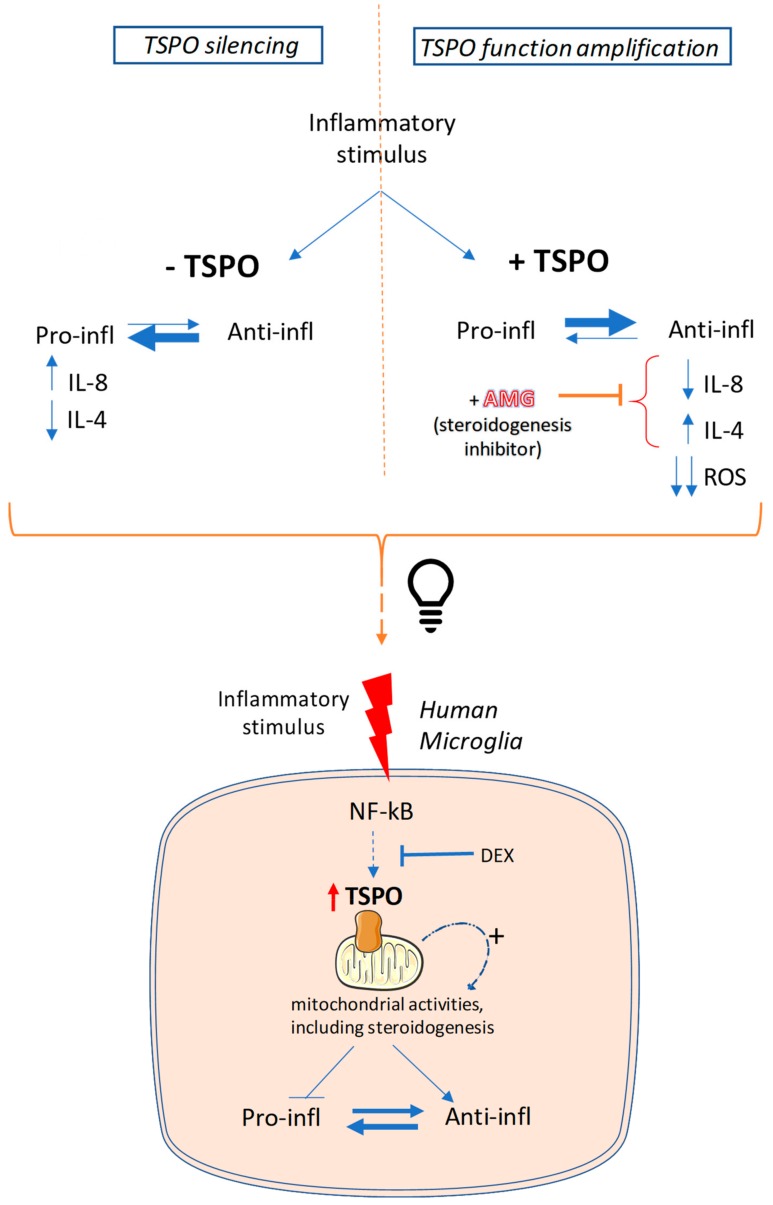
The suggested role of TSPO in orchestrating different cellular activities during inflammation of human microglia. In the above panel, the two experimental approaches are reported: (i) TSPO silencing (−TSPO) and (ii) TSPO function amplification by using TSPO ligands (+TSPO). In the -TSPO approach, the balance is shifted toward the pro-inflammatory phenotype (as indicated by the thicker arrow directed to the left). In the +TSPO approach, the balance is shifted toward the anti-inflammatory phenotype (as indicated by the thicker arrow directed to the right). This latter phenomenon is abolished when the steroidogenesis inhibitor aminoglutethimide (+AMG) is used. These observations indicate that the expression/activation of TSPO is related to the switch of microglial phenotype. The suggested hypothesis is outlined in the lower panel. The inflammatory stimulus activates the NF-κB pathway, which in turn could stimulate the TSPO overexpression, as a result of the binding of this transcription factor to the *TSPO* promoter. Our data suggest that TSPO is able to activate (+) specific mitochondrial activities, such as ROS production and neurosteroidogenesis, in order to counteract the pro-inflammatory state.

In conclusion, the obtained results suggested a TSPO role in orchestrating different cellular activities addressed to contrast the pro-inflammatory phenotype of human microglia, as schematic reported in Figure 8. TSPO negatively regulates the ROS production probably through the modulation of the mitochondrial activity. Moreover, the stimulation of neurosteroidogenesis by TSPO may activate a mitochondrion-to-nucleus pathway able to modulate the gene transcription of neurosteroid target genes (including IL-4 and IL-8) [3]. Finally, at plasma membrane level, the TSPO-stimulated neurosteroidogenesis products may potentiate the GABA_A_ receptor activity. Despite all these aspects shed light on some of the potential TSPO-mediated mechanisms, further studies are needed to deeply investigate both the molecular determinants of ROS modulation and the specific neurosteroids involved in genomic and not genomic activities.

## 4. Materials and Methods

### 4.1. Materials

Dulbecco’s Modified Eagle’s Medium, fetal bovine serum, penicillin, streptomycin, IL-1β, INF-γ, all TSPO ligands, and protease inhibitors were purchased from Sigma-Aldrich (Sigma-Aldrich S.r.l., Milan, Italy). All other chemical reagents were obtained from commercial sources.

### 4.2. Cell Culture

C20 human microglial cells were originally generated by David Alvarez-Carbonell, Ph.D. (Case Western Reserve University) [76].

C20 cells were cultured in DMEM-F12 medium supplemented with 10% fetal bovine serum (FBS), penicillin (100 U/mL), and streptomycin (100 mg/mL) at 37 °C in 5% CO_2_. The medium was supplemented with neomycin (600 µg/mL) as a selector of the immortalized cells expressing telomerase.

The short hairpin RNA C20 (pLKO-TSPO C20) cells were cultured in the same medium of C20 cells with the addition of blasticidin (2,5 µg/mL) for the selection of pLKO vectors. The TSPO knockdown in C20 cells was performed by the use of a lentiviral expression vector (pLKO-shTSPO). The TSPO knockdown lentiviral expression vector (pLKO-shTSPO) was constructed by replacing the original 1.9 kb stuffer in the pLKO.1 vector using AgeI/EcoRI restriction enzymes by a short hairpin double-stranded oligo using the following primers. 

shTSPO-F:

5’-CCGGCCACACTCAACTACTGCGTATCTCGAGATACGCAGTAGTTGAGTGTGGTTTTTG-3′

shTSPO-R:

5′-AATTCAAAAACCACACTCAACTACTGCGTATCTCGAGATACGCAGTAGTTGAGTGTGG-3′

pLKO.1-TRC cloning vector was a gift from David Root (Addgene plasmid #10878). The design of the human TSPO shRNA was taken from the RNAi Consortium [77] (Cambridge, UK) with the clone ID (TRCN0000060433). The scramble shRNA was a gift from David Sabatini (Addgene plasmid #1864). Twenty-four hours before transfection, 2.2 × 10^6^ HEK293T cells were seeded onto a 100 mm culture dish. Using a standard calcium phosphate transfection protocol, 10 μg of the pLKO.1-shTSPO vector, 7.5 μg of psPAX2 (Addgene plasmid #12260), and 2.5 μg of pMD2.G (Addgene plasmid #12259) plasmids were co-transfected into HEK293T cells. Virus-containing supernatants were collected 48 h after transfection, and were immediately aliquoted and stored at −80 °C. The titer of lentiviral preparation was determined with a colony formation assay using 2µg/mL puromycin selection and 0.1% Crystal Violet staining solution. Lentiviral transduction was performed by spinoculation at 2400 rpm for 60 min by adding a virus solution to cells at the multiplicity of infection of 35, which did not show toxic effects on the cells, in the presence of 8 µg/mL polybrene. Fresh C20 culture medium containing a 2 µg/mL of puromycin was added to cells 24 h after infection. The cells remained under selection until all the mock-transfected cells died. Surviving cells were pooled and cultured for further analysis.

### 4.3. Relative mRNA Quantification

C20 were seeded in a six-well plate (at density of 300.000 cells/well) in complete culture media for 24 h. Then, cells were exposed to mild (IL-1β 20 ng/mL) or strong (IL-1β 100 ng/mL + INF-γ 50 ng/mL) inflammatory stimulus in serum-free medium and incubated for 24 h. The relative mRNA quantification of TSPO, IL-6, IL-8, IL-4, and IL-10 genes was performed by real-time RT-PCR, as previously described [34]. In brief, total RNA was isolated using the RNeasy Mini Kit (Qiagen, Hilden, Germany). The purity of the RNA samples was determined by measuring the absorbance at 260:280 nm, using the NanoDrop Instrument (Invitrogen, Carlsbad, CA, USA). cDNA synthesis was performed with 500 ng of RNA using the iScript cDNA Synthesis Kit (Bio Rad, Hercules, CA, USA). The primers used for the RT-PCR were designed to span intron/exon boundaries to ensure that products did not include genomic DNA (Table 1). In the final real-time RT-PCR reactions, the forward and reverse primers (200 nm) and cDNA (50 ng) were added. All reactions were performed for 40 cycles using the following temperature profiles: 98 °C for 30 s (initial denaturation), 55 °C for 30 s (annealing), and 72°C for 3 s (extension). The beta-actin gene was used as the housekeeping gene. PCR specificity was determined using both a melting curve analysis and gel electrophoresis. Interleukins and TSPO mRNA levels for each sample were normalized against β-actin mRNA levels, and relative expression was calculated using the Ct value.

### 4.4. ROS Production Evaluation

The ROS generation was determined using the fluorogenic probe DCFH_2_-DA (Molecular Probes, Invitrogen). C20 WT cells were seeded in a 96-well plate (at density of 10.000 cells/well) and maintained in complete culture media for 24 h. Then, cells were incubated in PBS/glucose containing 25 µM DCFH_2_-DA for 30 min in the dark (37 °C). Then the medium was removed and replaced with PBS/glucose. In the presence of ROS species, DCFH_2_-DA is oxidized to fluorescent 2′,7′–dichlorofluorescein (DCF). The emitted fluorescence intensity of DCA was examined by using EnSightTM multimode plate reader with wavelengths of 485 nm (excitation) and 520 nm (emission). Then, cells were fixed with 3% paraformaldehyde for 20 min. At the end, cells were washed with PBS and incubated with crystal violet for 20 min at room temperature. After extensive washing, a solution of 1% SDS was added to each well for 1 h, and the absorbance at 595 nm was determined. The DCFH_2_-DA fluorescence values were normalized to the cell content of each well.

### 4.5. Western Blotting Analysis

C20 cells were seeded in P60 Petri Dishes at a density of 3 x 10^5^ cells. After 24 h, cells were stimulated with both mild inflammatory stimulus (IL-1β 20 ng/mL) and strong stimulus (IL-1β 100 ng/mL + INF-γ 50 ng/mL) in the presence or in absence of 1 µM DEX for 24 h. Then, cells were lysed by adding RIPA buffer (0.5% sodium deoxycholate PBS, pH7.4, 1% Igepal, 0.1% SDS, and protease inhibitors 4 µg/mL Apronitin, 1 µM Ortovanoate, and 0.1 mg/mL PMSF). The suspension were maintained at 4 °C for 2 h. Protein quantification in cell lysates was performed by a Bio-Rad DC Protein Assay by following the manufacture protocol. Cell protein extracts (20 µg) were diluted in Laemmli solution, resolved by SDS-PAGE (4–20%), transferred to PVDF membranes, and probed overnight at 4 °C with a primary anti-TSPO antibody (diluted 1:7500) obtained from the research group directed by Prof. Wetzel CH. The primary antibody was detected using anti-rabbit IgG light chains conjugated to peroxidase (COD. A6154 Sigma Aldrich S.r.l diluted 1:10,000). The peroxidase was detected thanks to a chemo-luminescent substrate (ECL, Perkin Elmer, Waltham, MA, USA), using the ChemiDoc™ XRS·+·instrument. Densitometric analysis of immunoreactive bands was performed using ChemiDoc™ XRS+ System (BioRad).

### 4.6. Predictive Analysis of Transcription Factor Binding to the Human TSPO Promoter

The TSPO promoter sequence was analyzed using Transcription Factor affinity Prediction (TRAP)_(http://trap.molgen.mpg.de) and PROMO (http://alggen.lsi.upc.es/cgi-bin/promo_v3/promo/promoinit.cgi?dirDB = TF_8.3) web tools. [78,79].

### 4.7. Cell Viability Assay

C20 WT cells were seeded in 96-well microplates at a density of 10,000 cells/well and maintained in their specific, complete culture media. The day after the seed, cells were treated with three different increasing concentrations (10 nM, 100 nM, and 1 µM) of TSPO ligands PK-11195, Ro5-4864, Etifoxine, and XBD-173 for 24 h. The cell viability was determined using the [3-(4,5-dimethylthiazol-2-yl)-5-(3-carboxymethoxyphenol)-2-(4-sulfophenyl)-2H-tetrazolium, inner salt] (MTS) assay according to the manufacturer’s instructions (Promega, Milano, Italy). This tetrazolium dye can be reduced by the metabolic reducing agents NADH and NADPH to a water-soluble formazan salt. The amount of formazan produced is considered to be a marker of cell viability [80]. The MTS reagent was added to TSPO ligand-treated cells and the colorimetric MTS conversion was quantified after 1 h by measuring the absorbance at 490 nm using the EnSightTM multimode plate reader, equipped with Kaleido Data Acquisition and Analysis Software.

### 4.8. Cytokine Production Quantification 

C20 cells were seeded in a 24-well plate (1 x 10^5^ cells/well) and maintained in their specific, complete culture media for 24 h. Then, the cells were pre-incubated with 100 nM of TSPO ligands (PK11195, Ro5-4864, Etifoxine, and XBD-173) in serum-free medium. Following 2 h of incubation, C20 cells were subjected to exogenous administration of mild inflammatory stimulus IL-1β (20 ng/mL) or a strong inflammatory stimulus (IL-1β 100 ng/mL plus INF-γ 50 ng/mL) and incubated for 22 h. The conditioned medium from each well was collected in order to quantify pro-inflammatory and anti-inflammatory cytokines. In the experiments performed to evaluate the specific contribution of TSPO ligands-induced steroids production, the cells were pre-treated (1 h before the addition of the TSPO ligands) with aminoglutethimide (AMG, 50 µM). The conditioned medium from each well was collected and centrifuged for 5 min at 20000× g (4 °C) before quantification of pro-inflammatory and anti-inflammatory markers. Pro-inflammatory and anti-inflammatory cytokines levels were quantified using a commercial enzyme-linked immunosorbent assay (ELISA). The most highly sensitive ELISAs were chosen to measure the concentrations of IL-8 (Cloud Clone Corp., detection range: 15.6–1000 pg/mL, sensitivity < 6.7 pg/mL), and IL-4 (Cloud Clone Corp., detection range: 15.6–1000 pg/mL, sensitivity < 5.6 pg/mL).

### 4.9. Statistical Analysis

The Graph-Pad Prism program (GraphPad Software Inc., San Diego, CA, United States) was used for data analysis and graphic presentation. All data are presented as the mean  ±  SEM. Statistical analysis were performed as indicated in each graphic. The *p*-value < 0.05 was considered to be statistically significant.

## Figures and Tables

**Table 1:** Nucleotide sequences, product size, and annealing temperature of the primers utilized in real-time RT-PCR experiments.

Gene	Primer Nucleotide Sequences	Product Size (Base Pair)	Annealing Temperature
IL-6	FOR: 5′-TCCTCGACGGCATCTCA-3′ REV: 5′-TTTTCACCAGGCAAGTCTCCT-3′	165 bp	55°C
IL-8	FOR: 5′-AAGAGAGCTCTGTCTGGACC-3′ REV: 5′-GATATTCTCTTGGCCCTTGG-3′	408 bp	56°C
IL-4	FOR: 5′-ACTTTGAACAGCCTCACAGAG-3′ REV: 5′-TTGGAGGCAGCAAAGATGTC-3′	74 bp	56°C
IL-10	FOR: 5′-CAAGCTGAGAACCAAGACCC-3′ REV: 5′-AAGATGTCAAACTCACTCATGGC-3′	141 bp	55°C
TSPO	FOR: 5′-CTTTGGTGCCCGACAAATGG-3′ REV:5′-CTGACCAGCAGGAGATCCAC-3′	51 bp	55°C
β-actin	FOR: 5′-GCACTCTTCCAGCCTTCCTTCC-3′ REV: 5′-GAGCCGCCGATCCACACG-3′	254 bp	55°C

## Supplementary Materials

Supplementary materials can be found at https://www.mdpi.com/1422-0067/20/18/4467/s1.

## Abbreviations

TSPO	Translocator Protein 18 KDa
KD	Knockdown
IL	Interleukin
LPS	Lipopolysaccharide
INF- γ	Interferon-γ
TNF-α	Tumor Necrosis Factor-α
VDAC	Voltage Dependent Anion Channel
CNS	Central Nervous System
CRAC	Cholesterol Recognition Aminoacidic Consensus
shRNA	Short Hairpin RNA
ROS	Reactive Oxygen Species
AMG	Aminoglutethimide
NF-κB	Nuclear Factor Kappa-Light-Chain-Enhancer of Activated B Cells
P450scc	Cytochrome P450 Side-Chain Cleavage
COX-2	Cyclooxygenase-2
TF	Transcription Factor
DEX	Dexamethasone
RE	Random Expectation
SCR	Scramble
TSS	Transcription Start Site
