# Supplementary material for: Microglial Pro-Inflammatory and Anti-Inflammatory Phenotypes Are Modulated by Translocator Protein Activation
Source: Int J Mol Sci. 2019 Sep 10;20(18):4467. doi: 10.3390/ijms20184467 (PMC6770267; doi:10.3390/ijms20184467)
Supplement: Supplementary file 1 [file ijms-20-04467-s001.pdf]

SUPPLEMENTARY DATA

Table S1. The table shows extracted p-values, Matrix IDs, Matrix names, and calculated 'corrected P_values' from the normalization file. The ranking is sorted form the lowest to the highest p-value, thus from the most probable to the less one. Data were obtained from the TRANSFAC database of transcription factors (http://transfac.gbf.de/TRANSFAC/).

Rank	p-value	Matrix_ID	Matrix_name
1	0.00682155638995285	M00194	V$NFKB_Q6
2	0.0109640281198492	M00261	V$OLF1_01
3	0.0166106667846406	M00975	V$RFX_Q6
4	0.0181658475001706	M00373	V$PAX4_01
5	0.0188015474876627	M00931	V$SP1_Q6_01
6	0.0193288686250781	M00255	V$GC_01
7	0.02031157683588	M01142	V$LRH1_Q5
8	0.0207937402731656	M01119	V$KAISO_01
9	0.0251588525533654	M01119	V$KAISO_01
10	0.0264585494546445	M00196	V$SP1_Q6
